# Water-soluble exudates from seeds of *Kochia scoparia* exhibit antifungal activity against *Colletotrichum graminicola*

**DOI:** 10.1371/journal.pone.0218104

**Published:** 2019-06-19

**Authors:** Adam J. Houlihan, Peter Conlin, Joanne C. Chee-Sanford

**Affiliations:** 1 USDA/ARS, Urbana, IL, United States of America; 2 Department of Crop Sciences, University of Illinois, Urbana, IL, United States of America; 3 Department of Natural Resources & Environmental Sciences, University of Illinois, Urbana, IL, United States of America; Tallinn University of Technology, ESTONIA

## Abstract

Plant seed exudates are composed of complex mixtures of chemicals with potential for bioactive compounds with antimicrobial properties. This study focused on kochia (*Kochia scoparia*), one of many weedy plant species considered invasive in many agricultural systems. Extraction of compounds in water yielded an exudate mass equivalent to 7% of the original seed mass used. Water-soluble exudates were tested against 16 known plant pathogens in disk diffusion assays and kochia exudates were found to inhibit *Colletotrichum graminicola*, the fungal causative agent of anthracnose and stalk rot in maize. The narrow range of fungi found as targets suggested the mechanism of inhibition may be specific rather than broadly antifungal. A decline in viability of cells over four orders of magnitude occurred within six hours of exposure to exudate. The minimum inhibitory concentration was 3.125 mg L^-1^. Hyphae formation in *C*. *graminicola* appeared inhibited following exposure to the exudate. Small molecular weight compounds as determined by GC/MS analysis showed high relative amounts of the sugars fructose, galactopyranose, glucose, and sorbitol, along with moderate proportions of organic acids and amino acids. Protein content averaged 0.7% in the standard concentration (100 mg mL^-1^) used for inhibition assays. Size fractionation of the exudate and subsequent disk diffusion assays revealed bioactive fractions with compounds in the MW range <5 kDa. To the best of our knowledge, this study is the first to show promising bioactivity against *C*. *graminicola* that was associated with water-extractable compounds from a common weed species. The results suggest that seeds of persistent plant species with long-lived seed banks like kochia may have potential for use in the discovery of compounds active in inhibiting fungal pathogens.

## Introduction

The seed stage of the plant growth cycle is critical to plant establishment [1], yet relatively few studies have investigated seed-derived chemicals beyond their important role in plant development. Seed exudates are known to have natural bioactive compounds that consist of complex mixtures of molecules [2], and are recognized for their protective role against soil pathogens during germination [3–5], affecting microbial growth [6–11], allelopathic interactions [12], and pharmaceutical and industrial product development [13–15]. In contrast to root exudate studies where the importance of chemicals in the interactions between plants, soil and microbes are well known [16–18], the significance of seed exudates in the spermosphere is still not well-studied and few investigations have been made on the extent of these bioactive compounds in plant species considered invasive or weedy.

The physical barrier of seed coats, along with chemical defenses such as antimicrobial secondary metabolites, form protective mechanisms against seed damage and potential pathogens until plants are germinated [19–21]. Moreover, some chemical antagonists leach from the seed surface to establish a protective zone of exclusion around the propagule [22]. Antimicrobial activity associated with seeds has often been attributed to phenolic acids, tannins, lignins, saponins or other small organic molecules that disrupt bacterial and fungal cell membranes [23–25]. This protection of seeds contributes to their longevity, leaving long-standing soil seed banks of many plant species. In agronomic systems, long-lived seed banks of invasive species are a major challenge for weed control measures. While seed-associated compounds have been studied for their direct role in protection for the host plant, fewer studies have been made on seed-derived chemicals as sources of natural antimicrobials with potential importance beyond the protection of the seed host.

The input of compounds made collectively by plants and seeds released to soils contribute to the pool of organic carbon essential to the growth and survival of the heterotrophic soil microbial community. These compounds rely on water for movement and bioavailability in biological interactions. The bioactive water-soluble components of seed exudates can exert either beneficial or antagonistic actions resulting in strategies for plant protection [26, 27] and the notion of disease suppressiveness in soils [28–30]. Weedy plant species, many with high fecundity and seed production, collectively contribute to large annual deposits of seeds, resulting in the so-called seed bank that can persist for years in some species [31–33]. These deposits of seed are a potential source of complex compounds and have been generally overlooked for their potential important contributions in biological relationships.

The goal of this research was to identify weed species that produce water-soluble seed exudates with antimicrobial properties. For study, we chose one of the most persistent weed species that produces a significant seed bank to focus our efforts. Kochia (*Kochia scoparia*) is a highly competitive summer annual species shrub in the family Chenopodiaceae [34, 35]. Due in part to its highly prolific seed production and tumbleweed-like dispersal, kochia has since become one of the most common agricultural weeds of the Great Plains states, reducing yields in several crops, including wheat, oats, and sugarbeets [36, 37]. A list of phytopathogens that affect economically important row crops were selected as the target organisms of the bioactive extractable compounds. In this study, we characterized the antifungal activity of water-soluble kochia seed exudates and low molecular weight chemical components of the mixture.

## Materials and methods

### Weed seed exudate preparation

Seeds from kochia (*Kochia scoparia*) were purchased commercially from Azlin Seed Service, Leland, MS. Standard commercial processing included three cycles of seed rinsing with distilled water to remove superficial residues, air-drying, and storage at 4°C until shipment. Following inspection to remove seeds with visible damage or deformity, water soluble seed exudates were prepared according to a similar procedure by Nelson and Hsu [38] using dry seeds (3 g) soaked in 30 ml of sterile ultrapure water for 24 h (25°C, dark). Seeds did not germinate under these conditions. Seeds were not sterilized prior to exudate preparation to avoid potential negative effects on the compounds exuded [39]. The resulting exudate was separated from the seeds by filtration through a fine wire mesh, filtered again through a 0.45 μm pore size filter under vacuum to remove small particulate matter, and then frozen at -80°C for 4 hrs. The exudate was then lyophilized for 16 h and the resulting dry mass was weighed. All crude exudates were resuspended in ultrapure water to a concentration of 100 mg dry mass mL^-1^, filter-sterilized (0.2 μm pore size) and stored at 4°C for further testing.

The rate of exudation was determined by placing a fixed mass of seeds (0.5 g) into water (5 ml) and collecting the exudate as described above following varying times (1, 4, 8, 16 h) of incubation. Exudate recoveries were also taken at interval times from a single separate aliquot of seeds (0.5 g) in water, starting with exudates recovered by filtration of the seeds after 1 h of incubation, adding fresh water, and repeating the cycle at 4, 8, and 16 h.

### Chemical analyses

#### Protein assay

The protein content in the resulting exudate was measured using the Pierce Microplate BCA Protein Assay Kit (Thermo Scientific) according to the manufacturer’s instruction. Absorbance was measured using a SpectraMAX 190 microplate reader at 562 nm. Protein concentrations were calibrated against a standard curve of bovine serum albumin (BSA) concentrations ranging from 25 to 2,000 mg mL^-1^.

#### GC/MS analysis

GC/MS analysis was used to identify relative quantities of low molecular weight compounds up to MW 4000 Da in the exudate using the services available through the University of Illinois Metabolomics Center. Briefly, exudates were dried and derivatized according to [40]: 90 min at 30°C (in 80 μl of 20 mg/ml methoxyamine hydrochloride in pyridine), followed by a 60 min treatment at 70°C with 80 μl MSTFA (N-methyl-N-trifluoracetamide). A volume of 1 ml was injected in a split ratio 10:1. The GC-MS system consisted of an Agilent 6890N GC, and Agilent 5973 mass selective detector and a HP 7683B (Agilent Inc, Palo Alto, CA, USA) autosampler. The chromatograms and mass spectra were evaluated for metabolite identification using the HP Chemstation (Agilent, Palo Alto, CA, USA) and AMDIS (NIST, Gaithersburg, MD, USA) programs. The data was normalized to the internal standard (hentriacontanoic acid) in each chromatogram and weight of exudate sample to obtain the relative concentration of each compound.

### Fungal cultivation and inoculum preparation

The kochia exudate was initially screened for inhibition of 16 fungal plant pathogens (Table 1), and results from this initial screening subsequently determined the focus on *Colletotrichum graminicola*. Cultures for all fungi used for screening and in subsequent studies with *C*. *graminicola* were routinely cultivated on potato dextrose agar (PDA) (Difco) or potato dextrose broth (PDB) (Difco) at 25°C for 72 h. Flocculation and hyphael clumping made it difficult to assess fungal growth by optical density, thus cell counts were performed using serial dilutions and standard plate count methods to calculate colony-forming units (CFU) per milliliter (cfu mL^-1^). Cultures were maintained on PDA plates by transferring aerial structures from the periphery of the growing culture to a new PDA plate with a flame-sterilized metal inoculation loop. Aerial structures from the growing culture were streaked across the entire agar surface and incubated at 25°C for 72 h.

**Table 1:** Inhibition of different fungal plant pathogens (10^4^ cfu ml^-1^) by kochia seed exudate.

Fungal Test Organism	Inhibition zone^a^ (diameter, mm)
*Colletotrichum graminicola*	23–27
*Taphrina deformans*	19–23
*Aspergillus flavus*	None
*Helminthosporium carbonum*	None
*Cercospora zeae-maydis*	None
*Cladosporium macrocarpum*	None
*Protomyces innundatus*	None
*Schizosaccharomyces japonicus*	None
*Entyloma ficariae*	None
*Pleospora herbarum*	None
*Mortierella verticillata*	None
*Rhisoclosmatium sp*.	None
*Spizellomyces pseudodichotomus*	None
*Spizellomyces kneipii*	None
*Rhizoctonia solani*	None
*Phytophthora sojae*	None

^a^Values are the range of zone sizes obtained in multiple assays conducted over the entire course of the study.

Fungal spore solutions were prepared according to guidelines established by the NCCLS [41]. Briefly, fungi were cultivated on the surface of PDA plates at 25°C for 72 h to allow substantial hyphael growth. Two ml of a sterile 0.85% NaCl solution were pipetted directly onto the plate and the fungal mass was gently probed with a flame-sterilized inoculation loop. The plate was then angled at 45° to allow the saline solution to pool, and 1 ml of the spore/saline solution was pipetted into a sterile tube. The spore/saline solution was enumerated by serial dilutions and plate counts. All preparations were then centrifuged (10,000 x g, 10 min, 4°C), washed with a 0.85% NaCl solution, centrifuged again, then resuspended to 10^5^ cfu mL^-1^ in 0.85% NaCl. This method yielded a standardized concentration of cells for use in all disk diffusion assays described below.

Cell morphological observations of *C*. *graminicola* cells was conducted by microscopy using a Nikon Eclipse 6800 microscope equipped with a Photometrics Cool Snap fx camera to observe changes to cell and hyphael morphologies following exposure to seed exudate. Liquid cultures of *C*. *graminicola* were exposed to three concentrations (0, 25, 50 mg mL^-1^) of kochia exudate and incubated at 25°C, then examined once daily for three days to observe any significant morphological differences.

### Antimicrobial disk diffusion assays

Agar disk diffusion was used as an *in vitro* method for evaluation antimicrobial activity following general standard procedures [42]. PDA plates were spread with 100 μl of the 10^5^ cfu mL^-1^ fungal spore solution and allowed to rest at room temperature for 1 hr. This brief room temperature incubation facilitated the absorption of excess inoculum liquid into the agar. Whatmann no. 3 filter paper disks (7 mm diameter) were sterilized by autoclaving for use in antimicrobial diffusion assays. Seed exudate (20 μl) was spotted onto the disks and allowed to air dry for 1 h. The disks were then transferred onto the inoculated PDA plates using flame-sterilized tweezers. The plates were incubated at 25°C for 72 h and diameters of zones of clearing in the fungal lawn were measured to provide a qualitative assessment of growth antagonism following exposure to the exudate.

Diffusion assays were also conducted following size fractionation of the seed exudate through a Pierce 10 ml D-Salt Dextran Desalting Column which has a size exclusion limit of 5 KDa. A 2.5 ml volume of 1XPBS suspension of kochia exudate was applied to a column and separated into twenty fractions by gravity flow (~125 μl fraction^-1^). Each fraction was lyophilized and weighed, then resuspended to a standardized volume for use in the disk diffusion assay with *C*. *graminicola*. Additionally, the protein content in each fraction was measured.

The effect of pH, temperature, detergent, and protease treatment on the anti-*Colletotrichum* activity of kochia seed exudates was assessed. After aqueous seed exudate was prepared as described above, 1 M HCl or 1 M NaOH was used to adjust the solution pH to 2.0 or 8.0. pH-adjusted exudates were incubated at 4°C for 24 h. Exudates were incubated for 24 hours at 25, 50, 75, and 100°C to assess thermal lability. To determine if the active compounds in kochia seed exudate was proteinaceous, 100 μL of autoclave sterilized 25% sodium dodecyl sulfate (SDS) was added to 900 μL of aqueous seed exudate and incubated at 4°C for 24 h. Exudates were also treated with 10 mg mL^-1^ pronase E (Roche) at 40°C for 24 h or 10 mg mL^-1^ proteinase K (New England Biolabs) at 20°C for 24 h. After each treatment, disk diffusion assays were conducted using *C*. *graminicola* as described above.

### Minimum inhibitory concentrations (MIC)

Since the relationship between clearing zone size and antimicrobial activity in disk diffusion assays is not linear, thereby limiting the determination of quantifiable effects, minimum inhibitory concentrations (MIC) were used to more accurately assess the antimicrobial activity in the seed exudates. A broth-based microdilution method was used according to the following procedure: PDB was inoculated with 10^**4**^ cfu ml^**-1**^ of each fungal species tested, and 50 **μ**l was added to the cells of a sterile 96-well plate. Serial microdilutions (2-fold increments) were conducted by adding 50 **μ**l of seed exudate (starting concentration = 100 mg crude dry mass mL^**-1**^) to the 50 **μ**l of inoculated PDB in each well. Final dilutions ranged from 50 mg mL^**-1**^ to 0.24 mg mL^**-1**^ of seed exudate. The 96-well plate was incubated at 25°C for 72 h and the wells were monitored for fungal growth using a spectrophotometric microplate reader. The minimum inhibitory concentration was calculated as the highest dilution in which fungal growth was not detected.

## Results

### Seed exudate recovery

The exudate mass recovered as a percentage of original seed mass was 7.18% (±0.9%) for kochia seeds with an average protein content of 0.7% in the standardized working stock of resuspended exudate (crude exudate mass standardized to 100 mg ml-1). The recovered mass of exudate from kochia seed was the highest compared with nine other weed species that ranged from 0.23–3.62% measured in preliminary studies. Increasing the amount of leaching time increased the quantity of exudate recovered with most of the yield obtained after 1 h incubation, however, cycled recoveries of exudate appeared to result in a higher overall crude extract mass (S1 Fig).

### Fungal inhibition

**Fig 1:**
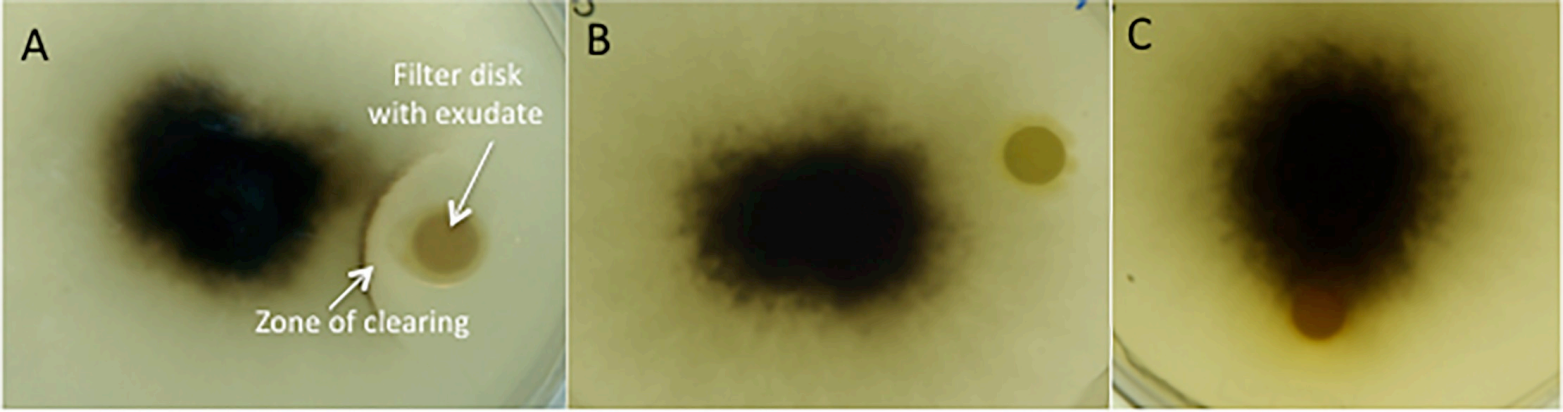
Representative images of *Colletotrichum graminicola* in agar disk diffusion assays with water soluble exudates of (A) kochia (*Kochia scoparia*), (B) velvetleaf (*Abutilon theophrasti*), and (C) giant ragweed (*Ambrosia trifida*).

**Fig 2:**
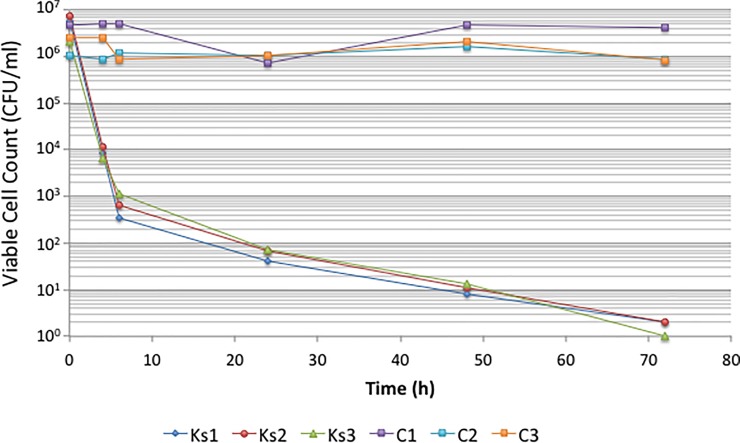
Inhibition of *C*. *graminicola* by kochia exudate (100 mg ml^-1^). Three independently extracted exudates were tested (Ks1, Ks2, Ks3). Control samples (C1, C2, C3) were untreated cells.

The kochia extract inhibited *C*. *graminicola* (23–27 mm diameter zone of clearing) (Table 1). *Taphrina deformans* was also inhibited by kochia exudate (19–23 mm diameter zone of clearing) but was not subject to further study here. *C*. *graminicola* did not exhibit any visible inhibition in disk diffusion assays performed in preliminary experiments with exudate from any other weed species (Fig 1). A rapid decline in viable cells of *C*. *graminicola* was measured following incubation in the presence of kochia extract, with four orders of magnitude cell loss occurring within the first six hours of exposure (Fig 2). Kochia seed exudates had a MIC value of 3.125 mg L^-1^ against *C*. *graminicola*.

**Fig 3:**
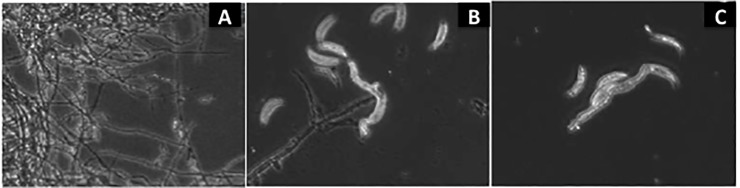
Phase contrast micrograph images showing vegetative growth and hyphael morphology of *C*. *graminicola* following incubation with kochia extracts. (A) untreated, 25°C, 72 h, (B) 25 mg mL^-1^, 25°C, 72 h, and (C) 50 mg mL^-1^, 25°C, 24 h.

Exposure of kochia exudates on *C*. *graminicola* exerted a distinct effect on their morphology (Fig 3). After 24 hours of incubation at 25°C regardless of exposure or not to exudate, evidence of germ tube (initial hyphae) development and hyphael growth can be seen in all cultures. At this point, branching of the hyphae is low and the majority of the *C*. *graminicola* cells are individual conidia (vegetative spores). After 48 h elapsed, branching of the hyphae in the untreated sample had increased and a greater percentage of the *C*. *graminicola* cells showed visible apical growth. When hyphae were treated with 25 mg mL^-1^ of exudate, budding appeared to cease and typical hyphael filaments became less visibly abundant. Samples treated with either 25 mg mL^-1^ or 50 mg mL^-1^ showed no significant signs of increased apical growth or branching. After 72 h elapsed, samples treated with 25 mg mL^-1^ and 50 mg mL^-1^ still showed no significant signs of increased apical growth or branching. Large aggregate masses of hyphae were observed in the untreated sample that appeared to be the beginnings of a mycelium as would be expected under normal growth conditions.

### Low molecular weight compounds in kochia exudates

All compounds detected in analyses were expected to be relatively low molecular weight, highly water soluble compounds. In earlier preliminary studies, seed exudates were also extracted by using organic solvents including ethylacetate, acetone, ethanol, methanol, and acetonitrile. None of the moderately polar solvents used in the extraction of kochia seeds resulted in inhibition of *C*. *graminicola*, and recoveries of dried exudates was typically 30-100X lower than by water extraction.

**Fig 4:**
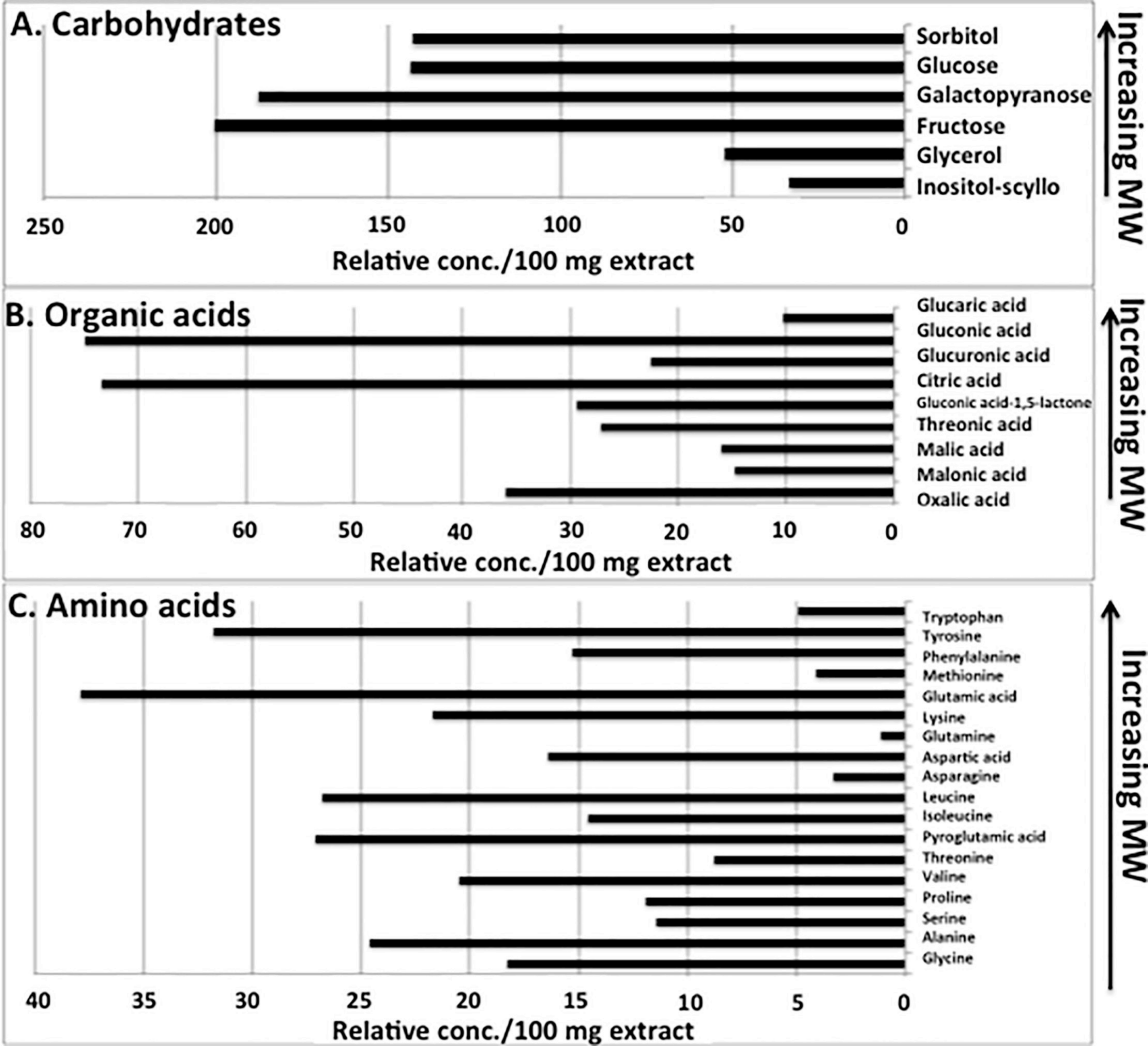
Low molecular weight compounds present in the highest relative amounts per 100 mg kochia exudate as detected by GC/MS analysis.

Analysis of low molecular weight compounds by GC/MS detected and identified 71 compounds in kochia exudate that could be grouped by general classification as organic carboxylic acids (MW range 74–457), amino acids (MW range 75–204), simple carbohydrates (primarily mono- and disaccharides, MS range 92–469), lipids (MW 282), phenolics (MW range 74–279), heterocyclics (MW range 112–244), and simple aliphatics (MW range 33–145). The highest relative concentrations of compounds were mainly sugars and sugar alcohols, followed by several organic acids and amino acids (Fig 4). Fructose, galactopyranose, glucose, and sorbitol were present in the highest relative concentrations among the sugars. Gluconic acid and citric acid were among the highest of the organic acids detected. Multiple amino acids were identified, with tyrosine and glutamic acid the highest in relative concentrations. Additional compounds identified in the exudate mix included additional lipids, aliphatics, phenolics, and heterocyclics, along with lower relative amounts of other sugars, organic acids, and amino acids (S2–S4 Figs).

When the pH of the kochia exudate was adjusted prior to use in disk diffusion assays with *C*. *graminicola*, loss of antifungal activity was observed (Table 2). Compared to untreated exudate, the most significant loss of activity was due to acidification (pH 2), which resulted in complete loss of bioactivity, however, buffering the pH back to pH 8 resulted in recovery of some activity. Heat treatment resulted in loss of activity, although some inhibition still occurred after heating exudates at 50°C. SDS detergent resulted in the loss of bioactivity, suggesting the possible role of proteins. To evaluate this further, proteolytic enzymes were used to treat the exudate, however, no loss of bioactivity appeared to occur using pronase E and proteinase K specifically.

**Table 2:** The effect of various chemical and physical treatments of kochia exudate on its antifungal activity against test organism *C*. *graminicola*.

Exudate treatment		Zone of clearing (mm)
**Untreated (control)**		24.33
	**pH 8.0**	22.10
**pH**	**pH 2.0**	0
	**pH 8.0 adjusted to pH 2.0**	8.32
	**pH 2.0 adjusted to pH 8.0**	11.66
	**25**	21.00
**Temperature (°C)**	**50**	17.00
	**75**	0
	**100**	0
**Detergent (2.5% SDS)**	**SDS + exudate**	7.50
	**SDS only**	7.70
	**Pronase E +exudate**	24.54
**Peptidases (10 mg/ml)**	**Proteinase K +exudate**	23.52
	**Peptidase only**	0

Size fractionation of the kochia exudate using column separation in a dextran matrix allowed a qualitative assessment to determine whether the bioactive fraction could be attributed to a threshold range of molecular size for which further chemical analysis could be done. The small volumes recovered of each fraction did not allow additional mass spectral identification to be performed, but protein concentration was measured (Table 3). All collected fractions contained protein, ranging from 0.004–0.237%, with the bioactive range restricted to fractions 3–9. There was no bioactivity associated with the void volume (≥5000 Da).

**Table 3:** Inhibition of *C*. *graminicola* measured by disk diffusion of kochia exudates following size fractionation.

Fraction	Zone of clearing (mm)	Relative protein content (%)
0	0	0.023
1	0	0.011
2	0	0.014
3	12	0.004
4	14.5	0.085
5	14	0.144
6	16	0.237
7	14	0.180
8	13.5	0.150
9	11.5	0.192
10	0	0.200
11	0	0.225
12	0	0.193
13	0	0.136
14	0	0.116
15	0	0.120
16	0	0.091
17	0	0.066
18	0	0.034
19	0	0.042
20	0	0.049

## Discussion

The inhibition of *C*. *graminicola* and *Taphrina deformans* by as yet unspecified water-extractable compounds from seeds of kochia is an intriguing finding that demonstrates a potential source of natural compounds with bioactivities of interest. The rather narrow spectrum of fungi affected by the kochia exudate suggests an antifungal mechanism that does not broadly affect other fungi. *C*. *graminicola* and *T*. *deformans* are two phylogenetically- and physiologically-unrelated fungal phytopathogens [43]. *C*. *graminicola* is a member of the Phyllachoraceae family and causes leaf anthracnose in maize and stalk rot [44–46], with other *Colletotrichum* species also noted as pathogens of fruit [47]. *Colletrotrichum* spp. has been listed as one of the top ten fungal pathogens to control [48, 49]. *T*. *deformans* is a member of the Taphrinaceae family that causes leaf curl in certain fruit trees [50], and while not further pursued in this study, the inhibition by kochia extract for this species also suggests a promising area for further study. It is not known if these fungi are pathogenic to *K*. *scoparia*. While numerous discoveries of natural compounds with antimicrobial activities have led to their pursuit as treatments for plant pathogens, e.g. [51–54], few studies have investigated weedy plant species and their seeds as potential sources for bioactive compounds.

The water-soluble characteristics of the compounds in the kochia exudate follow accordingly with the expectation that natural conditions in soils affecting the highest release of compounds may be attributed to higher moisture conditions and other environmental signals that may trigger seed germination. Kochia seeds appeared to exude compounds immediately after water saturation during the extraction procedure, and continued to release compounds up to 24 h before seeds could germinate. It is not known if the exudation of chemicals is always in equilibrium to the water content immediately surrounding seeds or if perhaps biotic interactions between seeds that could occur in a large depositional mass contribute to the quantity of chemicals exuded. Finding organic solvent-extractable compounds in separate exudate preparations that were not bioactive against *C*. *graminicola* suggested the inhibitory activity associated with the kochia extract was primarily associated with highly polar compounds that collectively extracted in a water matrix. Together, the effect of the co-extracted compounds extractable by water may have resulted in the bioactive mixture that allowed fungal inhibition observed in this study. Synergistic effects of the compounds tested in this work may also be possible, but this was not assessed in the present study.

The composition of the kochia exudate mixture had a surprisingly high relative content of several monosaccharide sugars, including fructose, galactopyranose, glucose, and sorbitol, along with lower relative amounts of other diverse saccharides. Sugars are often found as major chemical constituents in plant exudates, especially in roots [55]. While it is purely speculative at this point to know if sugars are directly associated with the antifungal bioactivity against *C*. *graminicola*, it is notable that the relative concentrations in the extracted pool of compounds is high. Certainly, the contribution of sugars toward the soil carbon pool is significant for recruitment of microbes that can be either beneficial or antagonistic to plants in the spermosphere, where initial stages of seed germination is a critical time for spelling successful seedling development. Corn seeds have been reported to release high concentrations of glucose, fructose, and sucrose [56], reportedly affecting bacteria in the spermosphere. One class of low molecular weight compounds detectable by GC/MS were phenolics, which are known to be involved in mechanisms of plant defenses in soil [57]. Phenolic compounds are also commonly released by plants and have been reported to have inhibitory effects on *Colletotrichum* spp. [47], but this class of compounds was not present in any significant quantities in the low molecular weight chemical mixture of the kochia exudate.

Amino acids were expected in the chemical composition of the mix along with a measurable protein content. Antifungal peptides have been reported and researched for their utility in plant-disease control [58–60] and most are in the range of 2–9 kDa. While speculative, the loss of bioactivity in the kochia exudate following SDS detergent treatment does suggest the possibility of protein involvement, although two types of peptidases did not appear to affect the fungal inhibition activity. If there are peptides associated with the bioactivity, the retention of bioactivity within a molecular size range tested using fractionated pools of exudate supported the involvement of low molecular weight compounds less than 5000 Da. The type and size of compounds in these fractions are much smaller than reported for average plant proteins [61]. The size range determination from this study allows a better strategy to purify fractions for characterization in future studies. Additional strategies to isolate protein fractions are warranted for future studies.

The morphological change in *C*. *graminicola* following exposure to kochia exudate provided some evidence for the physiological mechanism affected by the inhibition. The typical growth cycle of this fungus begins with a single spore, which following germination, forms an initial hyphael structure (germ tube) [62]. The hyphae grows apically, and branches frequently, eventually growing into a mass of hyphae or mycelium. The lack of hyphael development upon exposure to kochia exudate suggested that metabolic activity stopped and fungal reproduction had ceased. In general, chitin synthesis is essential for hyphael growth in filamentous fungi, and studies with *C*. *graminicola* have demonstrated the involvement of a chitin synthase and its role in virulence [46]. The inhibition to *C*. *graminicola* and *T*. *deformans* and not other fungal species by the kochia exudate suggest a specific mode of action as yet unidentified to certain fungi involving hyphae development for focus in future studies.

This study clearly showed the antifungal activity present in the kochia seed exudate. While the exact nature of the bioactive component in the kochia exudate is yet to be identified and are the subject of current efforts, this study provides a promising basis for further research to examine water soluble compounds associated with common weed species. Components of seed exudates may have pharmaceutical value and despite general high interest in natural bioactive compounds, there is a dearth of research in this area. More work is needed to isolate, identify, quantify, and determine the mechanism of action of antifungal compounds in *K*. *scoparia* exudate.

## Supporting information

S1 Fig Exudate recoveries from 5 g kochia seed batches (A) incubated for different amounts of time and (B) single batch where exudate was recovered at intervals by filtration and cycled again with addition of fresh water after each collection.
(TIFF)

S2 Fig Small molecular weight organic acids in ranges (A) & (B) present in relatively low amounts in 100 mg kochia exudate.
(TIFF)

S3 Fig Small molecular weight carbohydrates in different ranges (A) & (B) and amino acids (C) present in relatively low amounts in 100 mg kochia exudate.
(TIFF)

S4 Fig Small molecular weight lipids, short aliphatics, heterocyclics in different ranges (A) & (B) present in relatively low amounts in 100 mg kochia exudate.
(TIFF)
