# Supplementary figures and images for: Water-soluble exudates from seeds of Kochia scoparia exhibit antifungal activity against Colletotrichum graminicola
Source: PLoS One. 2019 Jun 19;14(6):e0218104. doi: 10.1371/journal.pone.0218104 (PMC6584005; doi:10.1371/journal.pone.0218104)

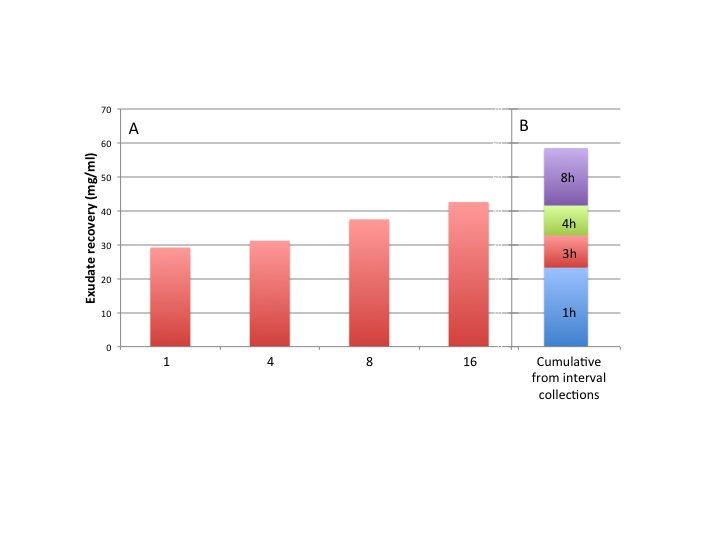

Supplement: S1 Fig — Exudate recoveries from 5 g kochia seed batches (A) incubated for different amounts of time and (B) single batch where exudate was recovered at intervals by filtration and cycled again with addition of fresh water after each collection. (TIFF) [file pone.0218104.s001.tiff]

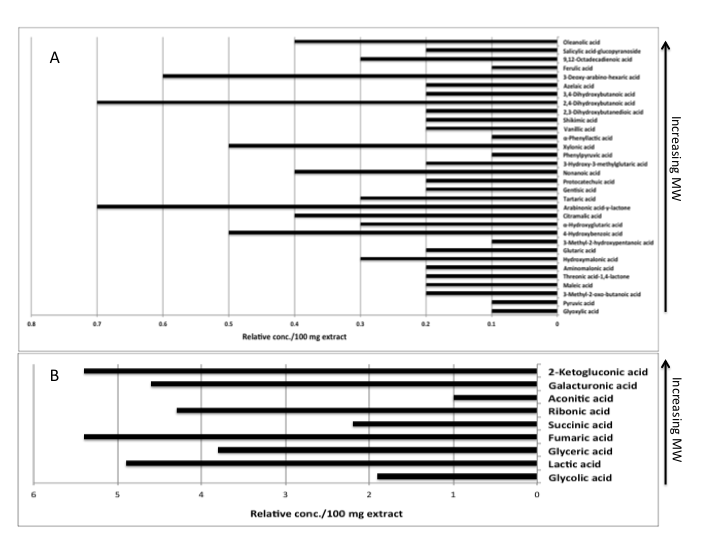

Supplement: S2 Fig — Small molecular weight organic acids in ranges (A) & (B) present in relatively low amounts in 100 mg kochia exudate. (TIFF) [file pone.0218104.s002.tiff]

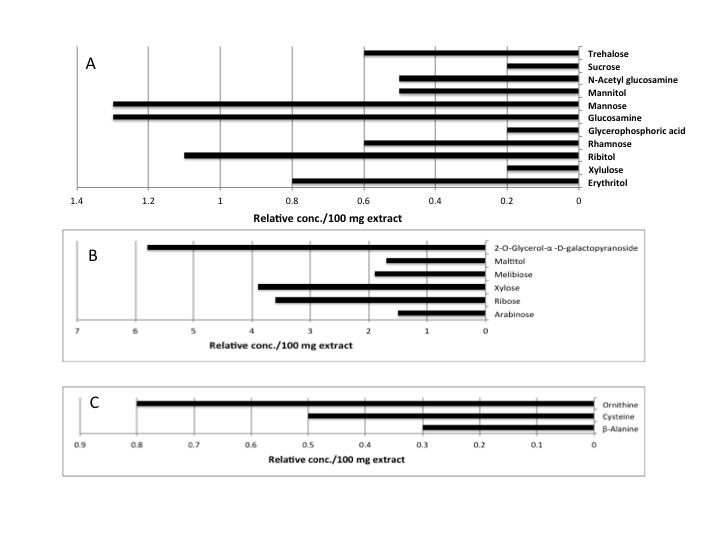

Supplement: S3 Fig — Small molecular weight carbohydrates in different ranges (A) & (B) and amino acids (C) present in relatively low amounts in 100 mg kochia exudate. (TIFF) [file pone.0218104.s003.tiff]

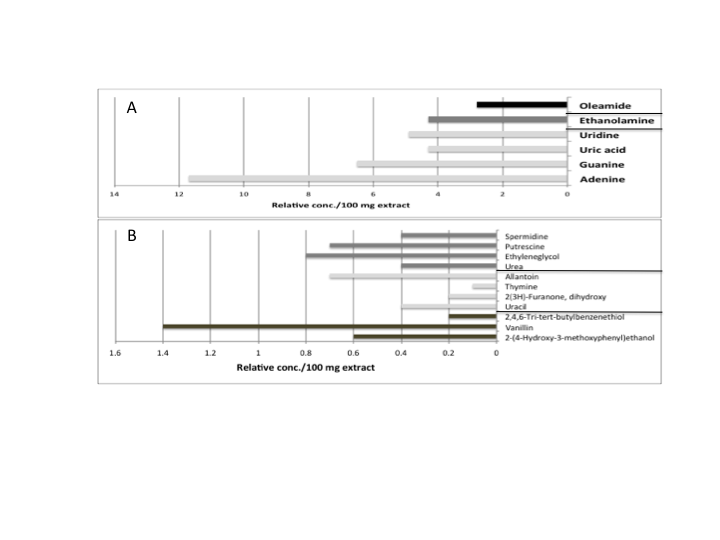

Supplement: S4 Fig — Small molecular weight lipids, short aliphatics, heterocyclics in different ranges (A) & (B) present in relatively low amounts in 100 mg kochia exudate. (TIFF) [file pone.0218104.s004.tiff]
